# Stressors and coping strategies among secondary school male students in Abha City, Saudi Arabia

**DOI:** 10.3389/fpsyg.2024.1303721

**Published:** 2024-02-07

**Authors:** Safar Abadi Alsaleem, Abdulaziz Hassan Al-qahtani, Essa Hasan Al-qahtani, Yahia Mater AlKhaldi

**Affiliations:** ^1^Department of Family and Community Medicine, College of Medicine, King Khalid University, Abha, Saudi Arabia; ^2^Family Medicine, King Khalid University, Medical City, Aseer, Saudi Arabia; ^3^Ministry of Health, Aseer, Abha, Saudi Arabia; ^4^Department of Research and Studies, Health Affairs, Aseer Region, Abha, Saudi Arabia

**Keywords:** chronic health problems, coping, secondary school students, stress, stressors, socioeconomic status, young age

## Abstract

**Background:**

Stress is a biological process in which exposure to stressors is associated with bad health effects, decreased ability of disease management, and a higher likelihood for disease-related complications and comorbidities. Stress has been considered the main factor in the etiology of many diseases such as diabetes mellitus, cardiac diseases, and body aches for centuries. Students meet many stressful situations such as the need for success, academic demands, homesickness, and lack of social support. Coping with stress can be a leading cause in preventing psychological distress and a serious illness.

**Methods:**

A correlational cross-sectional approach was applied to the current research. The research targeted all secondary school male students in Abha city. A multistage cluster sample was applied by selecting schools and students from the Directorate of Education in Abha. Self-administered questionnaire sheets were distributed to students in their classes. The questionnaire covered students’ sociodemographic data such as age, grades, parents’ education and work, living conditions, and comorbidities. Stressors to which the students were exposed were also enumerated with the degree of stress for each. Stress was assessed using the Holmes–Rahe Stress Inventory for youth. Coping skills were measured using the abbreviated version of the COPE Inventory.

**Results:**

The study included 324 students whose ages ranged from 15 to 20 years old with a mean age of 17 ± 0.9 years old. Approximately 33% of the students were in first grade; 44.4% of the students had no or minimal level of stress while major stress was recorded among 26.5% of the students. In total, 77.8% of the students had moderate coping and none had high coping ability.

**Conclusion:**

The study revealed that more than half of the students had significant levels of stress and one out of each four had major stress. The most common stressors were due to the school environment and future planning. Young age, low socioeconomic status, parents’ separation, and having chronic health problems were the most important determinants of stress among the students.

## Introduction

### Theoretical background

Stress is the physiological and psychological response to a stimulus that disrupts equilibrium (Myers and Sweeney, 2005). The stress process is impacted by social stratification structures, in which those in marginalized social and economic positions are more prone to encountering elevated levels of stress (Tiedt and Brown, 2014). It is an often-seen component of several emotions such as anxiety, frustration, wrath, worry, fear, sadness, and despair (Myers and Sweeney, 2005).

Stress manifests itself in physical symptoms that should not be overlooked. Some individuals may experience stress manifested as gastrointestinal discomfort, chest pain, or aberrant behavior such as jaw clenching (Myers and Sweeney, 2005). Stress has long been recognized as a primary cause of the development of various disorders, including diabetes mellitus, cardiovascular diseases, and bodily discomfort (Jiang et al., 2008). Moreover, it reduces the capacity for disease control and increases susceptibility to illness-related complications and comorbidities (Walters and Simoni, 2002; Walders-Abramson et al., 2014).

Stress is a common experience that everyone encounters from time to time. In the realm of academia, stress has become an unavoidable aspect of the lives of students and all those associated with them. Parents of adolescents must confront the burden of assisting their children in managing scholastic stress, in addition to their own stress (Park and Adler, 2003; Saat et al., 2010). For students, in particular, stress manifests in various situations. This can include adapting to a new school or college environment, facing the pressures of examination periods, navigating the complexities of forming new friendships, dealing with the illness of friends, coping with their parent’s divorce or separation, or experiencing the loss of a relative (Deng et al., 2022).

### Experimental backgrounds

Students frequently experience stress, including stress related to achieving success, managing time, and adapting to life and environmental changes, particularly during secondary school (Mohamed and Ahmed, 2012). The prevalence and severity of stress and depression among students in various academic disciplines are a cause for concern (El Ansari et al., 2014). A study in Kenya investigated stress levels, coping strategies, and mental health literacy among 400 secondary school students and found that stress in 66% was moderate, 31% was high, and 31% was low. A positive association was found between stress and avoidance coping strategies (Ayiro et al., 2023). Effectively managing stress can significantly reduce the risk of experiencing psychological distress and developing severe diseases (Kivimäki and Steptoe, 2018) including cancers (Dai et al., 2020). Coping tactics encompass several techniques, such as engaging in social interactions with friends and family, engaging in physical exercise, engaging in prayer, or proactively addressing the underlying causes of stress. These strategies are effective in promoting overall wellbeing (André-Petersson et al., 2006).

### The current study

Research conducted in the Middle East, specifically in Saudi Arabia, has documented the presence of psychiatric illnesses among teenagers and the contributing factors (Afifi et al., 2006; Khan et al., 2021; Alsaif et al., 2022). Moreover, the coronavirus disease 2019 (COVID-19) has affected various aspects of life, including life quality, sleep patterns, and increased anxiety and stress (Abd ElHafeez et al., 2022; Tahoun et al., 2023). In light of the established literature, it is hypothesized that male students in secondary schools in Saudi Arabia experience varying levels of stress. The sources of stress are expected to be diverse, including academic pressures, adapting to new environments, and managing interpersonal relationships. Furthermore, it is expected that the prevalence and severity of stress among students have increased, especially in the aftermath of the COVID-19 pandemic. The study aimed to explore the extent of stress experienced by male students and to identify the coping mechanisms employed to mitigate stressors. Additionally, it is hypothesized that effective stress management strategies, such as engaging in social interactions, physical exercise and addressing underlying stressors proactively, will contribute to overall wellbeing among male students in Saudi Arabian secondary schools.

## Materials and methods

### Methodology

The present research used a correlational cross-sectional approach, focusing on male students enrolled in secondary schools in Abha city, the administrative center of the Aseer region in southern Saudi Arabia.

A multistage cluster sampling method was employed in the selection of schools and students from the Abha Directorate of Education. The secondary schools for boys were distributed based on the geographical regions of Abha city. To ensure diversity, four distinct geographical areas were randomly chosen. Within each selected area, the largest governmental general secondary school for boys was included in the study. To further refine the sample, a simple random sampling technique was utilized within each selected school. This involved randomly selecting five classes, comprising one first-year class, two second-year classes, and two third-year classes. Formal approval was sought from the directorate of educational affairs in the region. Principals and teachers of the participant schools were informed about the objectives of the study before the implementation of the study. Hence, their active participation during data collection was ensured.

Using G * Power software, a minimum sample size of 321 participants was determined for this study based on assumptions of an effect size of 0.1, a power of 95%, an alpha error of 0.05, and a prevalence of stress set at 50%. In each selected class, all students were invited to participate in this study. In this study, the inclusion criteria included male students currently enrolled in secondary schools in Saudi Arabia, within a specified age range (e.g., 15 to 20 years). Additionally, participants must express willingness to take part in the study, possess the ability to comprehend and respond to survey/questionnaire items, and be available during the data collection period. Conversely, the exclusion criteria include female students, individuals not currently enrolled in secondary schools, those outside the specified age range, those lacking the willingness or ability to participate, and those with severe uncontrolled psychological problems. Furthermore, people with medical or psychological conditions that could significantly affect stress perception, if applicable, were excluded.

### The study instruments (data collection tools)

The questionnaire consisted of the following sections:

The first section collected sociodemographic information from the students, covering various aspects of their personal and familial background. The data included age categories, academic grade, parental education, parents’ occupation, monthly income, and family size. Additionally, information about the child’s birth order and student residence, familial dynamics, and living arrangements. The second section included an assessment of stress in youth. It was conducted using the Holmes–Rahe Stress Inventory. It has good internal consistency with Cronbach’s alpha level ranging from 0.81 to 0.86 for all domains (Noone, 2017). The participants in this study were asked to rank 43 life events based on a relative score, known as a Life Change Unit (LCU), which assigned different weights to each event based on its perceived impact on stress. The cumulative score, calculated by adding the points assigned to each event, served as an indicator of susceptibility to stress. A score of 150 points or less suggested a relatively low amount of life change and a correspondingly low susceptibility to stress-induced health problems. Scores between 150 and 300 points indicated approximately a 50% chance of experiencing a major stress-induced health problem in the next 2 years. A score of 300 points or more raised the odds to approximately 80%, as per the Holmes–Rahe prediction model, emphasizing the potential correlation between higher life change scores and increased susceptibility to stress-related health issues (Noone, 2017).

Brief-COPE, a 28-item self-report questionnaire, is designed to assess effective and ineffective coping strategies in response to stressful life events. The reliability of the assessment instrument is considered satisfactory, as indicated by Cronbach’s alpha values ranging from 0.5 to 0.9 across all domains. Coping, broadly defined as efforts to minimize distress associated with negative experiences, is a key focus. Widely used in healthcare settings, this scale aids in understanding how individuals emotionally navigate challenging circumstances, spanning a range of adversities, such as medical diagnoses, injuries, assaults, natural disasters, financial stress, or mental illness. Its application extends to counseling settings, providing valuable information about both helpful and unhelpful coping mechanisms. To calculate coping, individual scores for each domain were added together and expressed as a percentage of the maximum score (8 points for each domain). Individuals with a score below 50% were classified as having low coping abilities, while those with a score of 75% or more of the maximum score were classified as having excellent coping skills (Carver et al., 1989).

### Statistical analysis

Once the data had been gathered, it was reviewed, categorized, and input into the statistical software IBM SPSS version 22. The provided graphs were generated using the Microsoft Excel program. The statistical analysis was conducted using two-tailed tests and an alpha error level of 0.05. A *p*-value below 0.05 was deemed statistically significant. The frequency and percentage measures were employed to depict the distribution of students’ demographic information, stressors, stress levels, and coping mechanisms. The association between students’ stress levels and demographic factors, as well as coping strategies, was tested using the chi-square/Monte Carlo exact test and Fisher’s exact test.

### Ethical approval

This research received approval from the Joint Program of Family Medicine in the Aseer Region, the Research Ethical Committee at King Khalid College of Medicine, and the Director of Educational Affairs in the Aseer Region. This research adhered to the Declaration of Helsinki, which are ethical principles and guidelines for conducting medical research involving human participants. Ethical consent was obtained from parents for children under 18 years, while individuals aged 18 and above signed the consent forms themselves. The students were notified that their comments would be treated as completely secret and anonymous.

## Results

**Figure 1:**
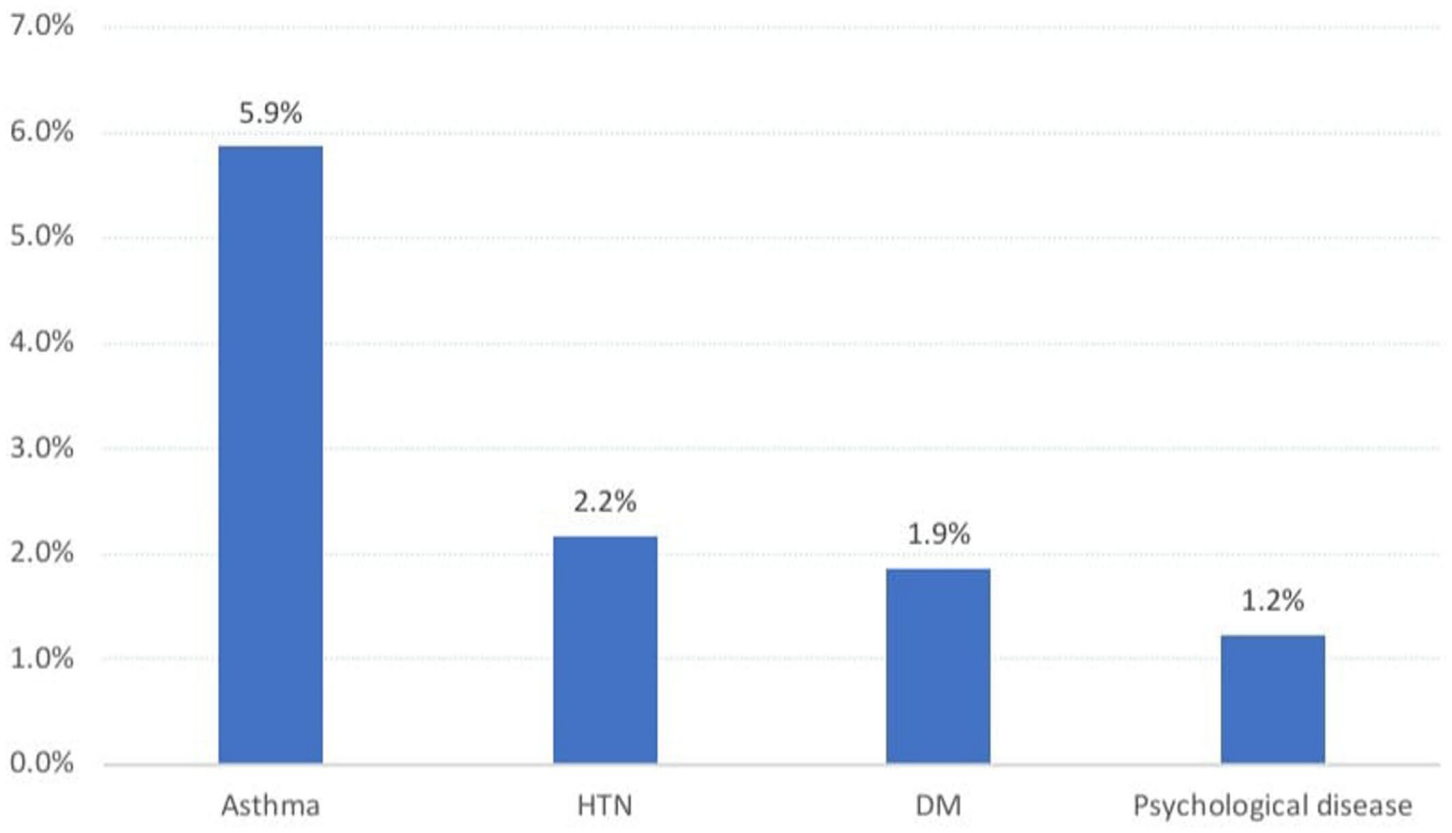
Chronic health problems recorded among sampled secondary school students in Abha city, Saudi Arabia.

The study had a total of 324 students, aged between 15 and 20 years, with a mean age of 17.0 ± 0.9 years. In total, 1.8% were in the third grade. With respect to parental statistics, 46.9% of the students’ fathers possessed a university degree, while 3.1% had no formal education. Furthermore, 40.7% of the pupils’ mothers possessed a university degree, whereas 13.3% had no formal education. Merely 5.6% of the pupils’ fathers were unemployed, and 24.1% were in a state of retirement, but 65.4% of their mothers were engaged in the occupation of being housewives. In terms of monthly income, 49.7% of the students’ families had a monthly income that exceeded 10,000 SR, whereas 21.3% had an income of less than 5,000 SR per month. In terms of family size, 19.1% of the students came from families consisting of 3 to 5 individuals, whereas 17.3% came from families with more than 10 members. Approximately 25% of the surveyed students were the eldest child in their family, whereas 5.6% were the youngest. In total, 82.4% of the students reside with both parents, whereas 6.5% live with their fathers and 11.1% live with their mothers (Table 1). Figure 1 depicts various disorders related to health. Approximately 10.0% of the students in the sample were diagnosed with asthma, 2.2% had hypertension, 1.9% had diabetes mellitus, and 1.2% experienced psychiatric disturbance.

**Table 1:** Sociodemographic data of sampled secondary school students in Abha city, Saudi Arabia.

Students’ sociodemographic data		No	%
Age in years	15–16	87	26.9%
	17–18	212	65.4%
	19–20	25	7.7%
	Mean ± SD	17 ± 0.9	
Grade	First	107	33.0%
	Second	114	35.2%
	Third (science)	103	31.8%
Father education	Illiterate	10	3.1%
	Primary	28	8.6%
	Middle	49	15.1%
	Secondary	85	26.2%
	University	152	46.9%
Mother education	Illiterate	43	13.3%
	Primary	48	14.8%
	Middle	33	10.2%
	Secondary	68	21.0%
	University	132	40.7%
Father work	Civil	125	38.6%
	Military	57	17.6%
	Private	46	14.2%
	Do not work	18	5.6%
	Retired	78	24.1%
Mother work	Housewife	212	65.4%
	Employee	83	25.6%
	Retired	29	9.0%
Monthly income	<5,000 SR	69	21.3%
	5,000–10,000 SR	94	29.0%
	>10,000 SR	161	49.7%
Family size	3–5	62	19.1%
	6–9	206	63.6%
	10+	56	17.3%
Child order	First	79	24.4%
	2–4	144	44.4%
	5+	83	25.6%
	Last	18	5.6%
Student residence	With parents	267	82.4%
	With father	21	6.5%
	With mother	36	11.1%

**Figure 2:**
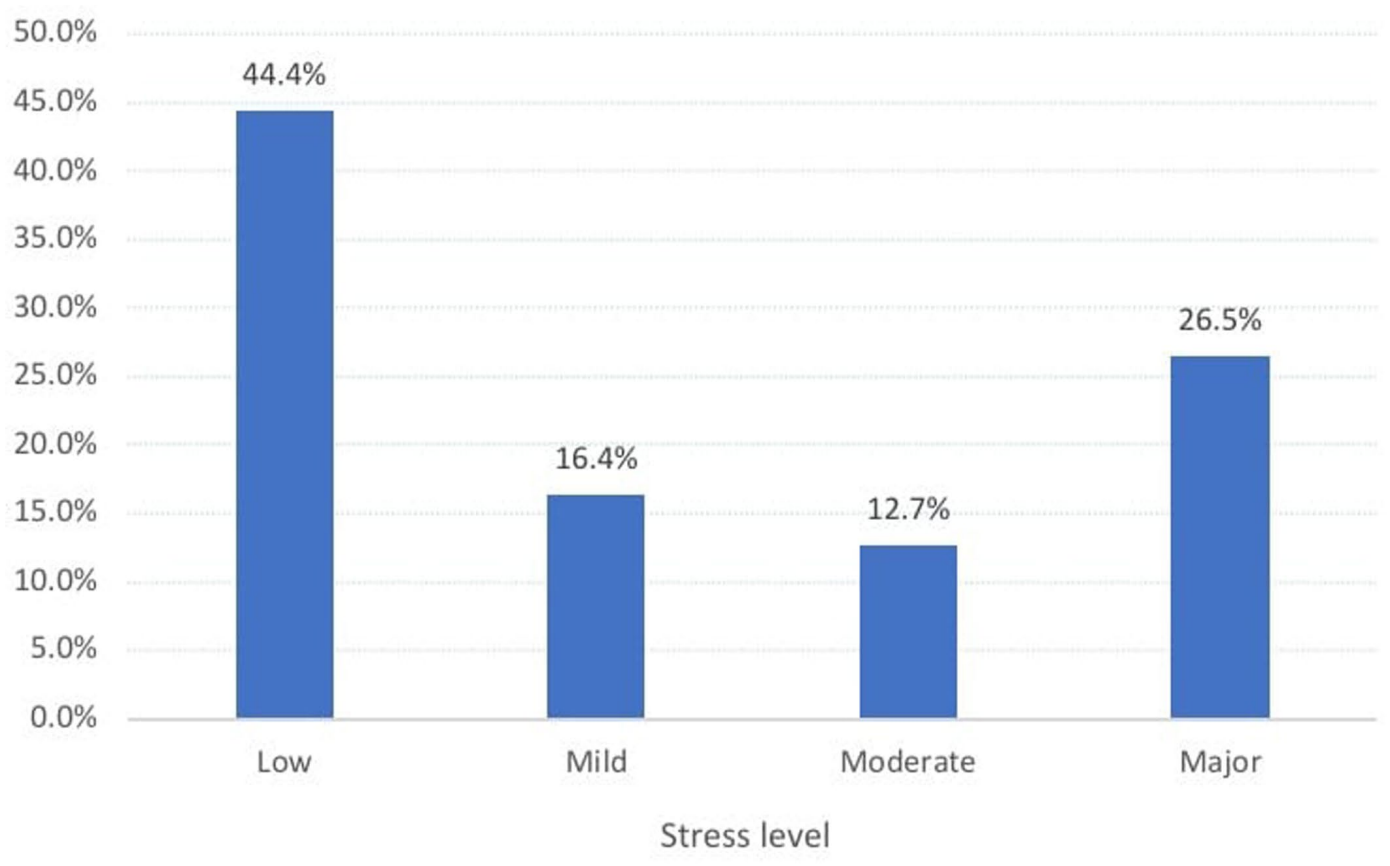
Level of stress among secondary school students in Abha city, Saudi Arabia.

Concerning the degree of stress, Figure 2 shows that 44.4% of the students experienced little or minimum stress, whereas 16.4% had mild stress, 12.7% had moderate stress, and 26.5% had major stress. In relation to stressors reported by the students. We found that 16.7% of the students experienced severe stress due to issues in the school environment, such as problems with teachers and academic matters. This was followed by future planning, which affected 16.4% of the students. Additionally, 11.1% of the students reported feeling a lack of control, whereas only 0.3% experienced severe stress due to troubled relationships with colleagues (Table 2).

**Table 2:** Stressors recorded among secondary school students in Abha city, Saudi Arabia.

Stressors	No		Moderate		Severe	
	No	%	No	%	No	%
Disturbed relations with parents	289	89.2%	14	4.3%	21	6.5%
Disturbed relations with brothers	287	88.6%	33	10.2%	4	1.2%
Suffered from school administration	228	70.4%	66	20.4%	30	9.3%
Suffered from teachers deal	212	65.4%	88	27.2%	24	7.4%
Disturbed relations with colleges	293	90.4%	30	9.3%	1	0.3%
Suffered from daily expense	285	88.0%	32	9.9%	7	2.2%
Suffered from the school environment	186	57.4%	84	25.9%	54	16.7%
Suffered from family income	282	87.0%	31	9.6%	11	3.4%
Suffered from future planning	184	56.8%	87	26.9%	53	16.4%
Suffered from a feeling of control	215	66.4%	73	22.5%	36	11.1%

Table 3 indicates that 8.3% of the students have a high level of adaptive coping mechanisms in terms of their ability to deal with challenges. The predominant form of adaptive coping observed was reliance on religious support (37.3%), followed by active coping (18.5%), and utilization of instrumental support (16.4%). Regarding maladaptive coping, a majority of 76.2% of the students exhibited a low degree of maladaptive coping. The most often reported strategy was self-blame, accounting for 17.6% of the cases, followed by self-distraction and denial, each accounting for 5.9%. Indeed, a significant majority of 77.8% of the children exhibited a moderate level of coping, whereas none demonstrated a high level of coping capacity.

**Table 3:** Coping of stress recorded among secondary school students in Abha city, Saudi Arabia.

Coping type	Coping domain	Low		Moderate		High	
		No	%	No	%	No	%
Adaptive coping	Active coping	126	38.9%	138	42.6%	60	18.5%
	Use emotional support	153	47.2%	147	45.4%	24	7.4%
	Use instrumental support	132	40.7%	139	42.9%	53	16.4%
	Venting	162	50.0%	141	43.5%	21	6.5%
	Positive reframing	125	38.6%	157	48.5%	42	13.0%
	Planning	114	35.2%	160	49.4%	50	15.4%
	Humor	175	54.0%	124	38.3%	25	7.7%
	Acceptance	131	40.4%	152	46.9%	41	12.7%
	Religion	92	28.4%	111	34.3%	121	37.3%
	Adaptive	126	38.9%	171	52.8%	27	8.3%
Maladaptive	Self-distraction	155	47.8%	155	47.8%	14	4.3%
	Denial	204	63.0%	101	31.2%	19	5.9%
	Substance use	291	89.8%	27	8.3%	6	1.9%
	Behavioral disengagement	224	69.1%	90	27.8%	10	3.1%
	Self-blame	118	36.4%	149	46.0%	57	17.6%
	Maladaptive	247	76.2%	77	23.8%	0	0.0%
Overall coping		72	22.2%	252	77.8%	0	0.0%

Table 4 demonstrates the association between students’ attributes and their ability to manage their stress levels. A statistically significant difference was seen between the high-stress levels of students aged 16 years or younger (53%) and those aged 17 to 18 years (32.1%). Furthermore, a significant disparity in stress levels was seen between first-grade children (54.2%) and third-grade students (32%). Regarding paternal education, there was a significant difference in stress levels between pupils with illiterate fathers (80%) and those with fathers who had a university education (42.8%) (*p* = 0.001). Furthermore, a significant disparity in stress levels was observed between students residing with their mothers (80.6%) and those living with both parents (32.2%) (*p* = 0.001). A significantly higher proportion of students with chronic health problems (73.0%) experienced higher levels of stress than healthy students (35.2%) (*p* = 0.001). Regarding the correlation between coping and stress levels, it was found that 63.9% of students with low coping skills had high levels of stress, whereas only 32.1% of those with moderate coping abilities had high levels of stress. The observed changes were determined to have a high level of statistical significance (*p* = 0.001).

**Table 4:** Relationship between students’ level of stress and their demographic data and coping in Abha city, Saudi Arabia.

Factor(s)		Stress level				*p*
		Low/Mild		Moderate/Major		
		No	%	No	%	
Age in years	15–16	41	47.1%	46	52.9%	0.001^*^
	17–18	144	67.9%	68	32.1%	
	19–20	12	48.0%	13	52.0%	
Grade	First	49	45.8%	58	54.2%	0.001^*^
	Second	78	68.4%	36	31.6%	
	Third (science)	70	68.0%	33	32.0%	
Father education	Illiterate	2	20.0%	8	80.0%	0.001^*^
	Primary	11	39.3%	17	60.7%	
	Middle	38	77.6%	11	22.4%	
	Secondary	59	69.4%	26	30.6%	
	University	87	57.2%	65	42.8%	
Mother education	Illiterate	21	48.8%	22	51.2%	0.130
	Primary	29	60.4%	19	39.6%	
	Middle	16	48.5%	17	51.5%	
	Secondary	47	69.1%	21	30.9%	
	University	84	63.6%	48	36.4%	
Father work	Civil	82	65.6%	43	34.4%	0.241
	Military	38	66.7%	19	33.3%	
	Private	25	54.3%	21	45.7%	
	Do not work	8	44.4%	10	55.6%	
	Retired	44	56.4%	34	43.6%	
Mother work	Housewife	131	61.8%	81	38.2%	0.617
	Employee	47	56.6%	36	43.4%	
	Retired	19	65.5%	10	34.5%	
Monthly income	< 5,000 SR	35	50.7%	34	49.3%	0.139
	5,000–10,000 SR	58	61.7%	36	38.3%	
	> 10,000 SR	104	64.6%	57	35.4%	
Family size	3–5	36	58.1%	26	41.9%	0.325
	6–9	122	59.2%	84	40.8%	
	10+	39	69.6%	17	30.4%	
Child order	First	52	65.8%	27	34.2%	0.233
	2–4	79	54.9%	65	45.1%	
	5+	53	63.9%	30	36.1%	
	Last	13	72.2%	5	27.8%	
Student residence	With parents	181	67.8%	86	32.2%	0.001^*^
	With father	9	42.9%	12	57.1%	
	With mother	7	19.4%	29	80.6%	
Chronic health problem	No	188	64.8%	102	35.2%	0.001^*^
	Yes	9	26.5%	25	73.5%	
Coping	Low	26	36.1%	46	63.9%	0.001^*^
	Moderate	171	67.9%	81	32.1%	

P: Pearson chi-square test. ^*^p < 0.05 (significant).

## Discussion

Stress is an inherent component of existence. Occasionally, it fulfills a practical function. Stress can serve as a driving force to propel you toward achieving a promotion at work or completing the final mile of a marathon (Britt and Jex, 2015). If someone fails to effectively manage stress and it persists over a prolonged period, it can significantly disrupt their professional responsibilities, family dynamics, and overall wellbeing (Bakker and Costa, 2014). This study aimed to explore the extent of stress experienced by male students and identify coping mechanisms. Effective stress management strategies, such as social interactions, physical exercise, and addressing underlying stressors proactively, contribute to overall wellbeing.

### The main study findings

The study found that 44.4% of the students experienced little or minimum stress, whereas 26.5% experienced major stress. The most often reported strategy for coping was religious support, followed by active coping, and utilization of instrumental support. A significant majority of 77.8% of the children exhibited a moderate level of coping. There was a significant difference in stress levels between students with different attributes such as age, paternal education, and family structure.

### Interpretation of the main findings of the study

Stress among secondary school pupils is marked by many intrapersonal changes, encompassing cognitive, emotional, and physical growth, as well as interpersonal changes, such as navigating relationships with classmates and family and adjusting to school transitions (Frydenberg, 2008). Adolescents encounter several transformations that might lead to heightened discomfort. Hence, it is crucial to comprehend the obstacles that adolescents encounter as distressing life events might pose a significant risk to their sound physiological growth and psychological welfare (McMahon et al., 2003; Grant et al., 2006).

In our study, we identified a high prevalence of stress among the pupils under examination. Similarly, a substantial prevalence of stress has been reported among secondary school students in various regions, including Kenya (3% low, 66% moderate, and 31% high) (Ayiro et al., 2023), Saudi Arabia (14.7 to 81.0%) (Almojali et al., 2017; Al-Shehri et al., 2022; Barnawi et al., 2023), Ethiopia (52.0%) (Nakie et al., 2022), and Malaysia (44.9%) (Wahab et al., 2013). Differences in the prevalence of stress across various studies can be attributed to several factors including variations in assessment tools, methodologies, and cultural contexts. By comprehending these variations, we can gain a better understanding of stress levels and recognize that it is a complex issue among secondary school students worldwide. It is worth noting that academic-related stress has been identified as a potential trigger for increased substance use (Boulton and O’Connell, 2017), disturbed sleep (Almojali et al., 2017), and reduced mental and physical health (Stults-Kolehmainen and Sinha, 2014) of young individuals during their academic journeys. Therefore, it is crucial to develop effective stress management skills that can positively impact the lives of young individuals and offer enduring benefits. This is particularly crucial as adolescence and early adulthood serve as a pivotal time for establishing long-term health-related behaviors and patterns, whether positive or negative. Therefore, focusing on enhancing academic stress-related coping abilities in young people during this critical developmental period is a key target (Sawyer et al., 2012; Pascoe et al., 2020).

The impact of environmental stresses on teenagers’ wellbeing is partially determined by their ability to utilize effective coping mechanisms, which can reduce feelings of distress (Nicolai et al., 2013). The coping methods acquired during this age have a significant impact on the developmental trajectories of young people, leading them toward either more or less adaptive paths. Furthermore, these coping strategies also serve as a prelude to the coping patterns that individuals will utilize throughout their adult lives (Compas et al., 2001). Evidence has shown that teenagers are aware of stress in their lives, and higher levels of stressors are linked to mental disorders (Grant et al., 2003).

Understanding the sources of stress and the coping techniques employed by adolescents is crucial due to the significant impact it has on their adaption, health, and development. The primary aim of performing this study was to achieve this objective. The primary objective of this research was to identify and analyze the various stresses encountered by secondary school students throughout the initial stages of adolescence, particularly those with limited life experience and a multitude of conflicting thoughts. Additionally, the study aimed to examine the coping mechanisms employed by these students in response to these stressors.

The present investigation revealed that approximately 55% of the students had clinically significant stress, with half of them reporting a high level of stress. These findings may be attributed to the participants’ youth, as most worried students were in first grade, where they encountered a new setting with unfamiliar classmates and activities. Furthermore, the sensation of assuming adult roles and the accompanying heightened sense of responsibility may serve as an additional rationale for these elevated stress levels. This hypothesis can be supported by the fact that the primary cause of stress was the school environment, which is unfamiliar to first-year students.

The fear of the future and the lack of clarity in sketching out future intentions were significant sources of stress. Exhibiting responsibility entails managing one’s emotions, which has been found to be the most prevalent source of stress among individuals of this age group, according to this study. An additional significant discovery made by this study is the strong correlation between a high perceived level of stress and the coping skills of students. It was evident that the students lacked effective coping mechanisms to manage stress and were unable to transform it into positive energy. Instead, they exhibited maladaptive coping strategies, particularly self-blame and self-distraction through activities such as watching TV or engaging with other media, which do not provide a lasting solution to the source of stress.

The highest category of stress was found among students from low socio-economic backgrounds, characterized by low parental education and low monthly income, as well as those living in families where one parent is absent due to death or separation. Furthermore, students who have chronic health conditions, regardless of the severity, experience significant stress. This stress negatively impacts their attitude toward any health issue, even small ones, due to their young age.

### Implication of this research

The study highlighted the high prevalence of stress among pupils and its association with various demographic factors including age, grade, parental education, living arrangements, and health conditions on stress levels. Furthermore, the study focused on the importance of incorporating culturally relevant coping resources in stress management programs. The research also highlights the correlation between demographic variables and stress levels, highlighting the need for targeted support. The study also emphasizes the importance of enhancing coping abilities, particularly among students with low coping skills. This information can guide educators, policymakers, and mental health professionals in implementing targeted measures to promote the wellbeing of students in the region.

### Strengths and limitations

The use of a multistage cluster sample contributes to a more representative selection of male students from diverse geographic areas in Abha city, increasing the generalizability of findings. The use of well-established tools such as the Holmes–Rahe stress inventory and the Brief-COPE questionnaire improves the reliability and validity of the study. Clear inclusion and exclusion criteria ensure that participants meet specific sex, age, enrollment status, and psychological wellbeing criteria. However, the study has notable limitations. The correlational cross-sectional design limits the ability to establish causation or understand the direction of relationships between variables, requiring caution in reaching definitive conclusions. Focusing solely on male students in Abha city limits generalizability to other regions and excludes insights into female experiences. Finally, the Holmes–Rahe stress inventory and the Brief-COPE questionnaire rely on self-report measures, which introduces the possibility of response bias. The reliance of the study on survey and questionnaire data may overlook the complexities of stress experiences, and the inclusion of qualitative methods could provide a more comprehensive understanding.

## Conclusion

The study demonstrated that over 50% of the students had a substantial degree of stress, while one in every four students experienced severe stress. The predominant pressures were attributed to the educational environment and anticipation of future endeavors. Religion was the most reported coping method in adaptive coping, whereas self-blame was the primary maladaptive coping approach. Approximately 75% of the students had intermediate coping skills. The primary factors contributing to stress among students were youth, low socio-economic position, parental separation, and chronic health conditions. Regular psychological assessments are necessary for students to promptly identify any potential effects of stress. Additionally, health education classes play a vital role in enhancing their ability to cope with stress. Furthermore, individuals should be educated on how to effectively utilize stressors as a kind of motivation and be vigilant for opportunities to enhance their performance.

Future research should consider longitudinal studies, comparative studies with different demographics or regions, qualitative investigations, interventional studies, academic performance metrics, cultural nuances, family support, the impact of technology-based interventions, and external factors such as the COVID-19 pandemic. Pursuing these avenues would help in understanding mental health among male students and guide the development of effective interventions.

## Data availability statement

The raw data supporting the conclusions of this article will be made available by the authors, without undue reservation.

## Ethics statement

The studies involving humans were approved by King Khalid University Ethical Committee. The studies were conducted in accordance with the local legislation and institutional requirements. Written informed consent for participation in this study was provided by the participants’ legal guardians/next of kin.

## Author contributions

SA: Conceptualization, Project administration, Resources, Supervision, Writing – review & editing. AA-q: Conceptualization, Data curation, Formal analysis, Software, Validation, Writing – original draft. EA-q: Validation, Writing – review & editing, Data curation, Formal analysis. YA: Conceptualization, Methodology, Project administration, Resources, Supervision, Validation, Visualization, Writing – review & editing.
